# Proinflammatory allogeneic dendritic cells enhance the therapeutic efficacy of systemic anti-4-1BB treatment

**DOI:** 10.3389/fimmu.2023.1146413

**Published:** 2023-08-15

**Authors:** Arwa Ali, Menghan Gao, Alexandros Iskantar, Hai Wang, Alex Karlsson-Parra, Di Yu, Chuan Jin

**Affiliations:** ^1^ Department of Immunology, Genetics, and Pathology, Science for Life Laboratory, Uppsala University, Uppsala, Sweden; ^2^ Chinese Academy of Science (CAS) Key Laboratory for Biomedical Effects of Nanomaterials and Nanosafety, CAS Center for Excellence in Nanoscience, National Center for Nanoscience and Technology, Beijing, China; ^3^ University of Chinese Academy of Sciences, Beijing, China; ^4^ Mendus AB, Stockholm, Sweden

**Keywords:** ilixadencel, allogeneic dendritic cells, α4-1BB therapy, tissue-resident CD8^+^ T-cells, tumor-reactive CD8^+^ T-cells, tumor-specific CD8^+^ T-cells

## Abstract

As an immune adjuvant, proinflammatory allogeneic dendritic cells (AlloDCs) have demonstrated promising immune-priming effects in several preclinical and clinical studies. The effector cells, including NK cells and T cells are widely acknowledged as pivotal factors in the effectiveness of cancer immunotherapy due to their ability to selectively identify and eradicate malignant cells. 4-1BB, as a costimulatory receptor, plays a significant role in the stimulation of effector cell activation. This study evaluated the anti-tumor effects when combining intratumoral administration of the immune-adjuvant AlloDCs with systemic α4-1BB treatment directly acting on effector cells. In both the CT-26 murine colon carcinoma model and B16 murine melanoma model, AlloDCs demonstrated a significant enhancement in the therapeutic efficacy of α4-1BB antibody. This enhancement was observed through the delayed growth of tumors and prolonged survival. Analysis of the tumor microenvironment (TME) in the combined-treatment group revealed an immune-inflamed TME characterized by increased infiltration of activated endogenous DCs and IFNγ^+^ CD8^+^ T cells, showing reduced signs of exhaustion. Furthermore, there was an augmented presence of tissue-resident memory (T_RM_) CD8^+^ T cells (CD103^+^CD49a^+^CD69^+^). The combination treatment also led to increased infiltration of CD39^+^CD103^+^ tumor-specific CD8^+^ T cells and neoantigen-specific T cells into the tumor. Additionally, the combined treatment resulted in a less immunosuppressive TME, indicated by decreased infiltration of myeloid-derived suppressor cells and Tregs. These findings suggest that the combination of intratumoral AlloDCs administration with systemic agonistic α4-1BB treatment can generate a synergistic anti-tumor response, thereby warranting further investigation through clinical studies.

## 1 Introduction

Our previous work has demonstrated that the intratumoral administration of proinflammatory allogeneic dendritic cells (AlloDCs) functions as an immune adjuvant (1, 2). This approach effectively modifies the immunosuppressive tumor microenvironment (TME) and successfully activates specific antitumor T cells (1, 2). The effectiveness of this concept has been extensively examined through numerous clinical studies, focusing on patients with hepatocellular carcinoma (3) gastrointestinal stromal tumors (GIST) (4) and renal cell carcinoma (4–6), with the drug candidate ilixadencel being specifically investigated. In addition, our recent work showed that intratumorally administration of AlloDCs can provoke a strong anti-tumor response when combined with systemic αCTLA-4 treatment in an αCTLA-4 monotherapy resistant model by unleashing a T-cell-dependent response (7).

Parallel to immune checkpoint blockade, agonistic agents acting on costimulatory receptors, for example 4-1BB, have been developed and evaluated in both preclinical and clinical research (8). 4-1BB, alternatively known as CD137, is a surface glycoprotein belonging to the tumor necrosis factor receptor superfamily. It is found on activated immune effector cells such as T cells and NK cells (9). Activation of 4-1BB triggers a diverse range of proinflammatory processes, including the generation of interferon-gamma (IFN-γ) and interleukin-2 (IL-2), the maintenance of immunological memory, and the enhancement of the cytolytic activity of antigen-specific primed T-cells through co-stimulation (9). 4-1BB stimulation can be mimicked by agonistic antibodies, resulting in strong immune responses against inoculated tumors in multiple experimental models (10, 11) and clinical settings. Importantly, the efficacy of α4-1BB agonistic antibodies could be enhanced by the specific priming of effector cells via cancer vaccines (12–15).

Given the fact that α4-1BB antibody relies on antigen-experienced activated T cells, and that intratumoral administration of AlloDC is aimed at prime tumor-specific T cells, we decided to study if the combination of these two immune-activating modalities could have synergistic anti-tumor effects.

In this study, we evaluated the combination therapy in two different tumor models, wherein AlloDC was generated from either C57BL/6NRj or BALB/c mice and injected into the other strain to mimic allogeneic situation. In both models, the local delivery of AlloDCs within the tumor demonstrated enhanced anti-tumor response when combined with systemic α4-1BB treatment. These findings underscore the need for additional research into the supplementary use of proinflammatory allogeneic DCs (ilixadencel) as an adjuvant therapy in patients undergoing α4-1BB antibody treatment.

## 2 Materials and methods

### 2.1 Tumor cell culture

For experiments conducted at Charles River (CR) Laboratories, the CT-26 murine colon carcinoma cells were obtained from the American Type Culture Collection (ATCC) and maintained at Charles River Laboratories Discovery Services. The CT-26 cells were cultured in RPMI-1640 medium supplemented with 10% (vol/vol) fetal bovine serum (FBS), 2 mM glutamine, 100 units/mL penicillin G sodium, 100 μg/mL streptomycin sulfate, and 25 μg/mL gentamicin.

For experiments conducted at Uppsala University, the CT-26 cells (American Type Culture Collection, ATCC) and the B16 cells were both maintained in RPMI-1640 medium containing 1 mM sodium pyruvate, 100 units/mL penicillin, 100 μg/mL streptomycin (1% PeSt), and 10% (vol/vol) heat-inactivated fetal bovine serum (FBS). All components were purchased from Invitrogen (Carlsbad, CA). The tumor cells were cultured in tissue culture flasks in a humidified incubator at 37°C with an atmosphere of 5% CO_2_.

### 2.2 Isolation and maturation of mouse bone marrow derived allogeneic DCs

The animal studies conducted in this research were approved by the Northern Stockholm Research Animal Ethics Committee under the reference number 5.8.18-19434/2019. To generate bone marrow-derived DCs for the CT-26 tumor model, the 8-10-week-old female wild-type (wt) C57BL/6NRj mice (H-2D^b^) were utilized. For the B16 tumor model, bone marrow-derived DCs were obtained from BALB/c mice (H-2D^d^). The mice were sourced from The Janvier Labs in France. The bone marrow cells were extracted by exposing the femur and tibia and flushing them out using a sterile syringe.

The harvested cells were cultured in IMDM supplemented with 10% heat-inactivated FBS, 1% PeSt, 1 mM HEPES, and 50 μM β-mercaptoethanol. All medium culture components were purchased from Invitrogen (Carlsbad, CA). The culture medium was supplemented with 20 ng/mL recombinant murine IL-4 and 20 ng/mL recombinant murine GM-CSF (Nordic BioSite, Sweden). Bone marrow cells were plated on non-treated Petri dishes (Sarstedt, Sweden). The medium was replaced every 3 days. On day 7 the non-adherent imDCs were collected. These imDCs were then treated with the COMBIG cocktail for 18 hours to induce maturation of the cells. The COMBIG cocktail consisted of 2.5 μg/mL R848 from InvivoGen (San Diego, CA), 20 μg/mL polyinosinic: polycytidylic acid (polyI:C) from Sigma-Aldrich (St. Louis, MO), and 1000 IU/mL IFN-γ from Nordic Biosite. The treated cells were cultured in a humidified incubator at 37°C with a 5% CO_2_ atmosphere.

### 2.3 Animal experiments part 1

The experiment was carried out at Charles River (CR) Laboratories, which served as the contracted service provider. The production of AlloDCs occurred at the Uppsala University Lab. Charles River Laboratories Discovery Services adheres to the guidelines set forth in the Guide for Care and Use of Laboratory Animals, ensuring proper practices in animal restraint, husbandry, surgical procedures, regulation of feed and fluids, as well as veterinary care. Furthermore, the animal care and use program at CR Discovery Services has obtained accreditation from the Association for Assessment and Accreditation of Laboratory Animal Care International (AAALAC), confirming compliance with established standards for the care and use of laboratory animals.

#### 2.3.1 CT-26 tumor model

For the in vivo therapeutic experiment, 9-week-old female BALB/c mice (H-2D^d^) were used. These mice were obtained from Charles River Laboratories in the United States. To initiate the experiment, the mice were subcutaneously injected on the right hind flank with 3x10^5^ CT-26 murine colon cancer cells suspended in PBS. The mice then received intratumoral (i.t.) vaccinations with AlloDCs derived from C57BL/6NRj mice (H-2D^b^). Mice received two i.t. injections of PBS or 1x10^6^ AlloDCs on days 14 and 21 after tumor implantation. The frozen AlloDCs were thawed, washed, and re-suspended in mouse plasma with 10% DMSO (Invitrogen) before injection. Respective groups received α4-1BB (BioXcell clone LOB12.3) on days 14 (2.5 mg/kg) and 21 (0.1 mg/kg) intraperitoneally (i.p.). Mice were sacrificed when the tumor volume exceeded 1500 mm^3^ or if bleeding ulcers developed. Tumor volume was calculated by the formula: Volume = length × width^2^ × π/6. Mice that exhibited tumor regression in the α4-1BB groups were subjected to a re-challenge with an equivalent amount of CT-26 on day 72, and their tumor growth was monitored and tracked in these mice.

### 2.4 Animal experiments part 2

The experiments were conducted at Uppsala University, and the animal studies were approved by the Northern Stockholm Research Animal Ethics Committee under the reference number 5.8.18-19434/2019.

#### 2.4.1 B16 tumor model

The 9 weeks old female C57BL/6NRj mice (H-2D^b^) were used *in vivo* therapeutic experiment (Charles River Laboratories, United States). The mice were subcutaneously injected on the right hind flank with 1x10^5^ B16 murine melanoma cancer cells suspended in 100 µL of PBS. AlloDCs derived from BALB/c mice (H-2D^d^) were administered to the mice intratumorally (i.t.). The mice received two i.t. injections of either PBS or 1x10^6^ AlloDCs on days 6 and 12 after tumor implantation. The frozen AlloDCs were thawed, washed, and re-suspended in mouse plasma with 10% DMSO (Invitrogen) before injection. The respective groups also received α4-1BB (BioXcell clone LOB12.3) intraperitoneally (i.p.) on days 6 (2.5 mg/kg) and 12 (0.1 mg/kg). Mice were euthanized if their tumor volume exceeded 1500 mm^3^ or if bleeding ulcers developed. Tumor volume was calculated using the formula: Volume = length × width^2^ × π/6.

#### 2.4.2 Depletion

The 6-week-old, female Balb/c mice (The Janvier Labs) were subcutaneously inoculated on the right hind flank with 3x10^5^ CT-26 tumor cells in 100 µl of DPBS (Invitrogen).

Mice received the same treatment as *in vivo* therapeutic animal experiments. CD4 depletion antibody (In Vivo Mab αmouse CD4, clone GK1.5, BioCell) and CD8 depletion antibody (In Vivo Mab αmouse CD8a, clone 2.43, BioCell) were injected (i.p) on day 13, 14, 15, 20, 21 and 22. Mice were sacrificed when the tumor volume exceeded 1500 mm^3^ or if bleeding ulcers developed. Tumor volume was calculated by the formula: Volume = length × width^2^ × π/6.

#### 2.4.3 Survival analysis

Individual animals were closely monitored for tumor growth throughout the study until reaching humane endpoints or until their tumor volume surpassed the study endpoint volume (EPV) of 1500 mm^3^. Tumor size was determined using the formula: volume = length × width^2^ × π/6. The time to endpoint (TTE) was calculated to assess the time for each mouse to reach the endpoint. The TTE was determined using the equation: TTE = [log(EPV) - b]/m, wherein b represents the intercept, and m represents the slope of the line obtained from linear regression analysis of a log-transformed dataset of tumor growth over time. The dataset included the first measured tumor volume when the EPV was exceeded and the three consecutive measured tumor volumes immediately preceding the attainment of EPV. In the case of any mice that died from treatment-related causes, their TTE value was set equal to the day of their death. Animals that died from non-treatment-related causes were excluded from the analysis. To analyze the survival data, a survival curve was generated using the Kaplan-Meier method, and the Log-Rank (Mantel-Cox) test was employed to compare the survival curves.

#### 2.4.4 Characterization of tumor and organs

Female BALB/c mice obtained from the Janvier Labs, aged 6 weeks, were subcutaneously inoculated with 3×10^5^ CT-26 tumor cells into the right hind flank using a 100 μl volume of DPBS (Invitrogen). The mice were subjected to the same treatment protocol as described in the in vivo therapeutic animal experiment. On day 26, the mice were euthanized, and their tumor tissues and spleens were collected for subsequent analysis.

### 2.5 Analysis of CD45^+^ tumor-infiltrating cells

#### 2.5.1 NanoString

Following the aforementioned tumor establishment and treatment procedure, the harvested tumor samples were subjected to the enzymatic digestion using Liberase™ (Roche, Solna, Sweden) before total RNA was isolated using the RNeasy® Plus RNA isolation kit (Qiagen AB, Sweden). The gene expression levels were directly measured as mRNA counts using the Mouse-Pan cancer immune-oncology kit (NanoString, Seattle, USA). Gene expression analysis was performed using nSolver Analysis software (NanoString).

#### 2.5.2 Flow cytometry analyses of the tumor microenvironment

After the tumor samples were harvested as previously described, they were processed to obtain single-cell suspensions through enzymatic digestion using Liberase™ from Roche (Solna, Sweden). CD45^+^ cells were then isolated using mouse MojoSort™ CD45 isolation beads from Miltenyi Biotec (Germany). Subsequently, the isolated cells were stained using antibodies specific to the T-cell panel, myeloid cell panel, and tissue-resident memory panel, with the specific antibodies listed in **Supplementary Table S1**. The stained cells were analyzed using a CytoFLEX LX flow cytometer from Beckman Coulter Life Sciences (Brea, CA). The flow cytometry data were visualized and analyzed using Partek® Flow® software, version 10.0, from St. Louis, United States.

### 2.6 Peptides restimulation assay

H-2L^d^ restricted gp70 peptides and beta-galactosidase peptides (as irrelevant control) were purchased from Biosite. Splenocytes were collected following the treatments. Approximately 1×10^5^ cells were re-stimulated with each peptide in triplicate at a concentration of 20 µg/mL in a 96-well plate, and incubated for 3 days. The supernatant from the cultures was collected and subjected to analysis for mouse IFN-γ levels using the ELISA (Mabtech, Nacka Strand, Sweden).

### 2.7 Statistics

The data were presented as mean values accompanied by the standard error of the mean (SEM). Statistical analysis was conducted using GraphPad Prism software, version 9.0, from La Jolla. For comparisons involving more than two groups, the nonparametric one-way analysis of variance (ANOVA) test was employed. Kaplan-Meier survival curves were generated and compared using the Log-Rank test. Values with P<0.05 were considered to be statistically significant. All detailed P value is reported in **Supplementary Table S2**.

## 3 Results

### 3.1 AlloDCs enhanced the therapeutic efficacy of α4-1BB antibody that dependent on CD8^+^ T-cells

**Figure 1:**
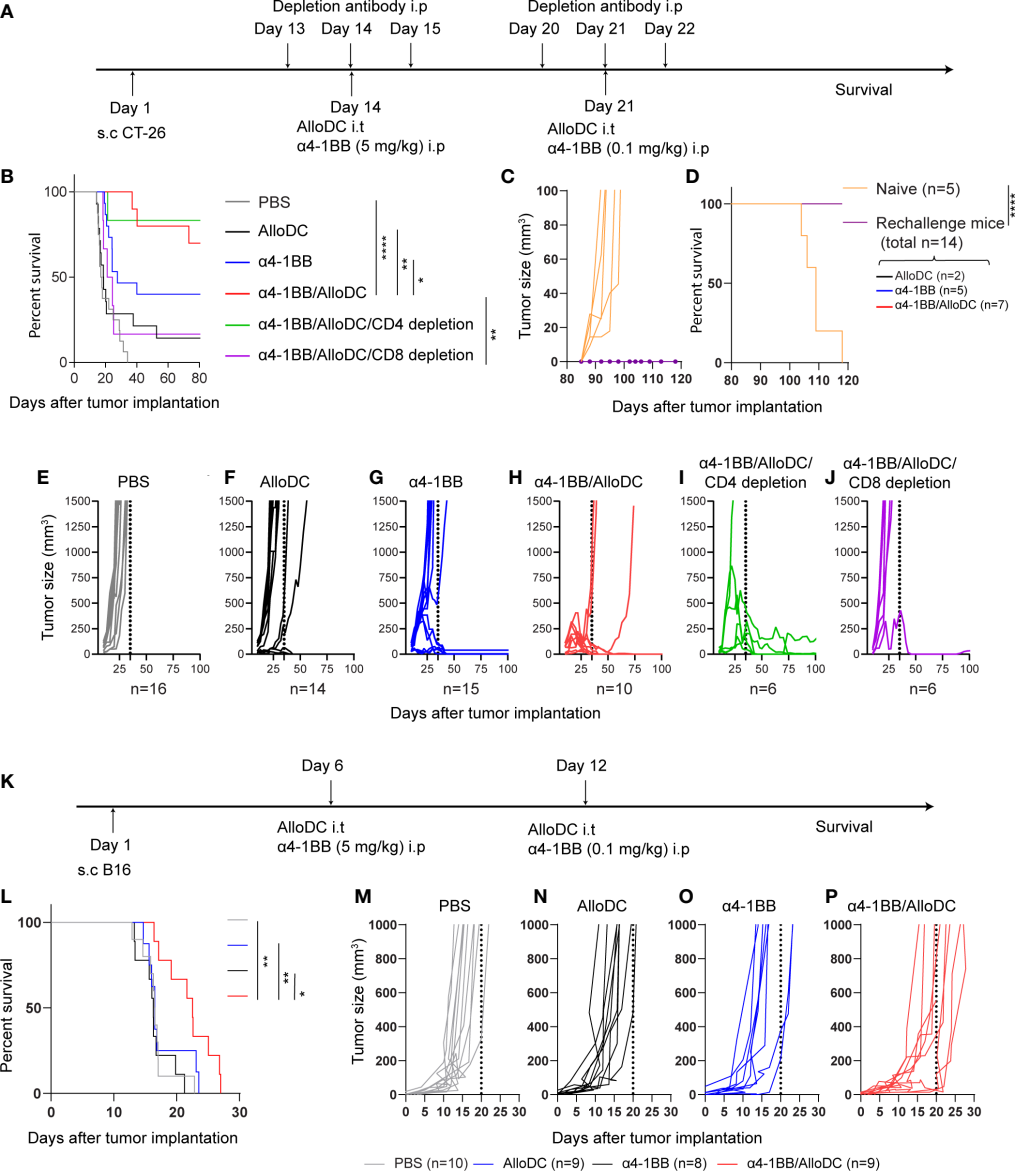
AlloDCs enhanced the therapeutic efficacy of α4-1BB antibody that dependent on CD8^+^ T-cells. **(A)** Schematic illustration of the experiment outline of the CT-26 model. **(B)** Mouse survival (Kaplan-Meier curve) in the CT-26 model after different treatments as indicated (PBS n=10, AlloDC n=14, α4-1BB n=15, α4-1BB/AlloDC n=10, α4-1BB/AlloDC/CD4 depletion n=6, α4-1BB/AlloDC/CD8 depletion n=6). **(C, D)** Tumor size in individual mouse and survival after re-challenge with CT-26 tumor. All mice that survived in **(B)** were re-challenged and none of them got tumor after re-challenge. The number of rechallenged mice from each group are labeled in figure panel. **(E–J)** Tumor size in individual mouse in different treatment groups as in **(B)**. **(K)** Schematic illustration of the experiment outline of the B16 model. **(L)** Mouse survival (Kaplan-Meier curve) in the B16 model after different treatments as indicated. (PBS n=10, AlloDC n=9, α4-1BB n=8, α4-1BB/AlloDC n=9). **(M–P)** Tumor size in individual mouse in different treatment groups as in **(L)**. The size of each group (n) is labeled in each panel below. All survival curves were compared using the Log-Rank test. (*P<0.05, **P<0.01, ****P<0.0001).

To examine the therapeutic efficacy of AlloDCs combined with 4-1BB therapy, we treated BALB/c mice (H-2D^d^) bearing subcutaneous syngeneic colon tumors with AlloDCs generated from C57BL/6NRj mice (H-2D^b^) administrated intratumorally in combination with systemic α4-1BB treatment (**Figure 1A**). As monotherapy, AlloDC or α4-1BB only cured a minority of mice (14% and 30%, respectively, of mice with complete response). However, 70% of the mice rejected the tumor after the combined treatment with both AlloDC and α4-1BB (PBS VS α4-1BB/AlloDC P<0.0001, AlloDC VS α4-1BB/AlloDC P=0.0013, α4-1BB VS α4-1BB/AlloDC P=0.0394) (**Figures 1B, E–H**). Complete responders were re-challenged at day 80 and it was observed that all mice successfully rejected the newly inoculated tumors. The comparison between naïve mice and mice subjected to tumor rechallenge showed a significant difference (P<0.0001), as depicted in **Figures 1C, D**. This finding suggests the generation of a memory anti-tumor immune response. Together, these data showed the enhanced therapeutic efficacy and the long-term protection conferred by this combination therapy.

In addition, we assessed the role of different T-cell subsets in eliminating the tumor cells by depleting either CD4 or CD8 T-cells in the combined-treatment group (**Figure 1A**). CD8-depletion, but not CD4-depletion, abolished the efficacy of α4-1BB/AlloDC treatment (α4-1BB/AlloDC VS α4-1BB/AlloDC CD8 depletion P=0.0031) (**Figures 1B, H–J**), thus demonstrating that CD8^+^ T-cells are indispensable for the therapeutic efficacy of α4-1BB/AlloDC treatment.

In order to generalize the concept, we also examined the therapeutic efficacy of AlloDCs combined with α4-1BB therapy on C57BL/6NRj mice (H-2D^b^) bearing subcutaneous murine B16 melanoma tumor and treated these mice with AlloDCs generated from BALB/c mice (H-2D^d^) (**Figure 1K**). As monotherapy, AlloDC or α4-1BB treatment didn’t show any therapeutic effect. On the other hand, the α4-1BB/AlloDC combination therapy delayed the tumor growth and prolonged the mice survival (PBS VS α4-1BB/AlloDC P=0.0042, AlloDC VS α4-1BB/AlloDC P=0.0009, α4-1BB VS α4-1BB/AlloDC P=0.0493) (**Figures 1L–P**). These data demonstrated that AlloDCs as an immune adjuvant, their therapeutic potential is not restricted by mouse strain.

### 3.2 AlloDCs boost the lymphoid compartment signature for α4-1BB/AlloDC therapies

**Figure 2:**
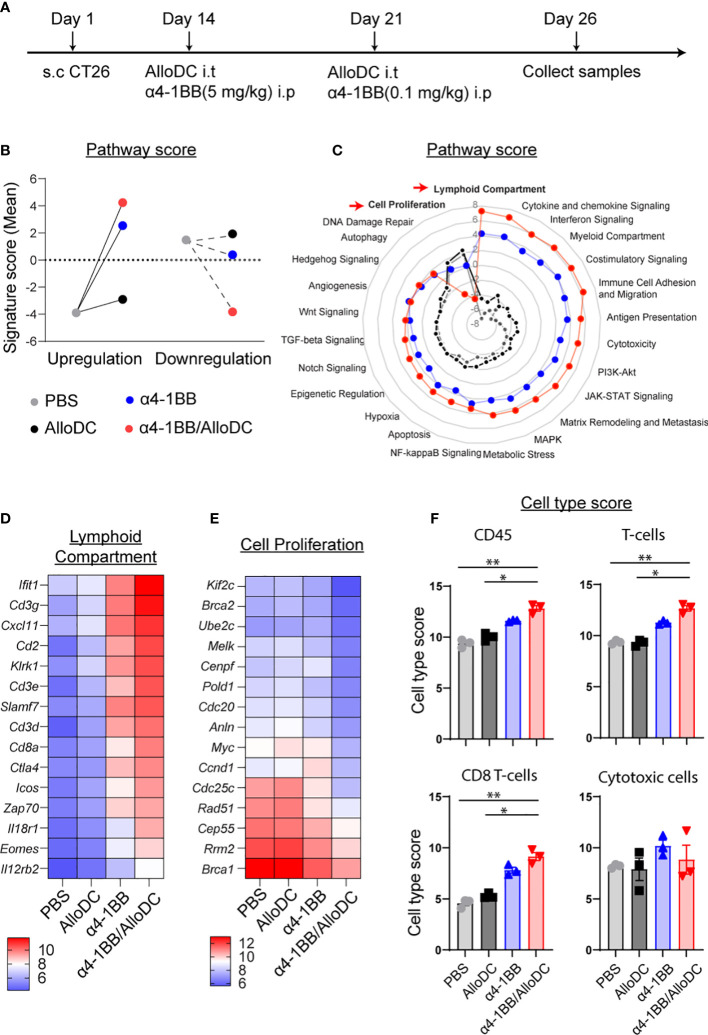
AlloDCs boost the lymphoid compartment signature for α4-1BB/AlloDC therapies. **(A)** The experimental setup is depicted in a schematic illustration. **(B)** The comparison between the PBS-treated group and the grouped samples based on up- or down-regulation is presented as the sum of pathway signature scores determined from NanoString mRNA profiling. **(C)** The radar map displays the pathway scores from different treatment groups. **(D, E)** The heatmap showcases the top 15 differentially expressed genes from lymphoid compartment pathways and cell proliferation. **(F)** The abundance of tumor-infiltrating immune cells in each mouse from different treatment groups is presented as cell-type scores from NanoString mRNA profiling. Error bars represent SEM and the mean values were compared using one-way ANOVA nonparametric test. (*P<0.05, **P<0.01).

Subsequently, we conducted Nanostring RNA profiling of the TME following the treatment in order to gain insights into the underlying mechanisms behind the therapeutic benefits (**Figure 2A**). Through gene set analysis, we identified notable differences in pathway signatures between the treatment groups (**Figure 2B**). These findings provide valuable information about the potential molecular mechanisms involved in the observed therapeutic effects. Compared to the PBS control treatment, α4-1BB monotherapy and combination treatment groups significantly reversed the signature score distribution across the zero line (**Figure 2B**). When looking into detailed pathways signatures, compared to PBS treatment group, AlloDC monotherapy did not substantially affect the pathway signatures. However, both α4-1BB monotherapy and α4-1BB/AlloDC combination therapy drastically shifted the pathway signatures. It is clear that in the combination treatment group, the signature associated with cell proliferation was radically reduced, and the signature associated with lymphoid compartment was drastically increased. Genes that are classified in these 2 pathways are listed in **Figures 2D, E**. The lymphoid compartment signature classified genes highly associated with activated T cells (*Cdc3e*, *Cdc3g*, *Cdc3d*, *Cd8a*, *Zap70*, *Icos*, *Ctla4*, *Eomes*) indicating a high infiltration of T cells in the combination treatment group. The cell proliferation signature classified genes associated with cell cycle, DNA repair, indicating dying of the cancer cells. These data reflected changes in the TME after different treatments and the distribution of pathway score, which might explain why the addition of AlloDC to α4-1BB improved the therapeutic outcome. Furthermore, a higher number of tumor-infiltrated immune cells were present in the combination group, including total CD45^+^ cells, T-cells, CD8^+^ T-cells (CD45^+^ cells PBS VS α4-1BB/AlloDC P=0.0031, AlloDC VS α4-1BB/AlloDC P=0.0315; T-cells PBS VS α4-1BB/AlloDC P=0.0092, AlloDC VS α4-1BB/AlloDC P=0.0127, CD8^+^ T-cells PBS VS α4-1BB/AlloDC P=0.0022, AlloDC VS α4-1BB P=0.0415) (**Figure 2F**).

### 3.3 α4-1BB/AlloDCs combined treatment confers more CD8^+^ T-cell infiltration with activated phenotype coupled with enhanced DC activation

**Figure 3:**
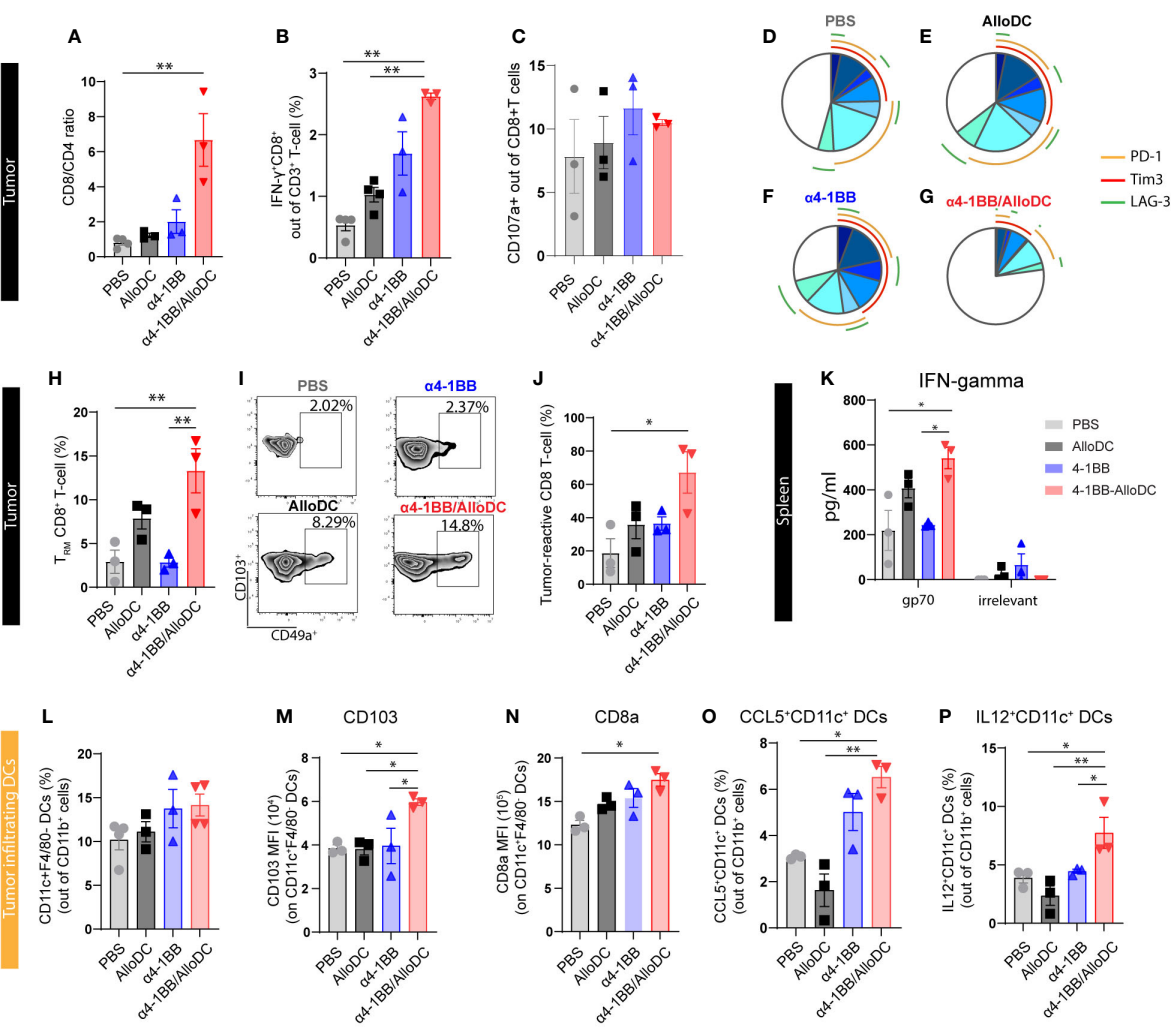
AlloDCs combined with α4-1BB treatment attract CD8^+^ T-cell populations with activated phenotype by enhanced DC activation. **(A)** The ratio of CD8+ and CD4+ T-cells in the tumor-infiltrating T-cell population (gated as CD3+ T-cells) is analyzed using flow cytometry. **(B)** The percentage of IFNγ^+^CD8^+^ T-cells in the tumor is measured. **(C)** The percentage of CD107a^+^CD8^+^ T-cells in the tumor is determined. **(D–G)** The percentage of phenotypically exhausted CD8+ T-cells in the tumor is assessed, represented as triple, double, and single positive for PD-1^+^, Tim3^+^ and LAG3^+^ markers on CD8^+^ T cells. **(H)** Percentage of tumor infiltrating tissue-resident memory (T_RM_) CD8^+^ T-cells (gated as CD49a^+^CD103^+^ T-cells out of CD69^+^CD8^+^ T-cells) in each treatment group, analyzed by flow cytometry. **(I)** Representative density plot showing tumor infiltrating T_RM_ in each treatment group. **(J)** Percentage of tumor-reactive CD8^+^ T-cells (CD39^+^CD103^+^ out of CD8^+^ T-cells) in tumor samples from different treatment groups. **(K)** IFN-γ expression level in the supernatant of *in vitro* cultured splenocytes, harvested from each treatment group and re-stimulated with either gp70 peptides or non-relevant peptides. **(L)** Percentage of the tumor-infiltrating DCs (CD11C^+^F4/80^-^ out of CD11b^+^CD45^+^ cells) in each treatment group. **(M, N)** The expression of CD103a and CD8a on the tumor infiltrating DCs. **(O, P)** Percentage of the CCL5^+^ and IL12^+^ tumor-infiltrating DCs in each treatment group. Error bars represent SEM and the mean values were compared using one-way ANOVA nonparametric test. (*P<0.05, **P<0.01).

Based on the aforementioned data, it can be inferred that the combination of AlloDC and α4-1BB treatment led to the activation of lymphoid compartment pathways. This observation prompted us to assess the status of tumor-infiltrating T cells. Notably, we observed a general increase in the CD8/CD4 ratio, indicating a higher proportion of CD8^+^ T cells compared to CD4^+^ T cells in the combination therapy groups. Additionally, there was a higher presence of IFN-γ-producing CD8^+^ T cells in the combination therapy groups compared to the monotherapy groups. However, the expression of CD107a, a marker of cytotoxic degranulation, did not show significant differences between the CD8^+^ T cells in the combination therapy groups and the monotherapy groups (CD8/CD4 ratio PBS VS α4-1BB/AlloDC P=0.0014, IFN-γ^+^ PBS VS α4-1BB/AlloDC P=0.0014 AlloDC VS α4-1BB/AlloDC P=0.0022) (**Figures 3A–C**). Moreover, among the CD8^+^ T-cells from α4-1BB/AlloDC group, there were significantly fewer PD-1-, Tim3-, and/or LAG-3-positive cells indicating less phenotypic exhaustion (**Figures 3D–G**).

We next sought to further dissect the profile of CD8^+^ T-cells with a focus on distinct tumor-controlling subtypes, including tissue-resident memory T-cells (T_RM_) (16), tumor-reactive CD8^+^ T-cells (17), and antigen-specific T-cells (18) which have been reported to contribute to the success of cancer immunotherapy. We identified that in α4-1BB/AlloDC combination groups, T_RM_ (CD8^+^CD49a^+^CD103^+^CD69^+^) cells were remarkably higher compared to monotherapy groups (T_RM_ PBS VS α4-1BB/AlloDC P=0.0174, α4-1BB VS α4-1BB/AlloDC P=0.0217) (**Figures 3H, I**). In the combination therapy groups, there was a notable increase in CD8^+^ T-cells exhibiting a tumor-reactive CD39^+^CD103^+^ phenotype (PBS VS α4-1BB/AlloDC P=0.0127) (**Figure 3J**). Additionally, an increase of CD69 on CD8 T-cells was observed in the tumor-draining lymph nodes (**Supplementary Figure 1**). Furthermore, since the CT-26 tumor cells express the endogenous retroviral antigen gp70, we were able to investigate the T-cell response specific to this neoantigen. Following stimulation with the gp70 peptide, splenocytes collected from the combination treatment groups demonstrated significantly higher secretion of IFN-γ, confirming the establishment of a neoantigen-specific CD8^+^ T-cell response. (IFN-gamma PBS VS α4-1BB/AlloDC P=0.0127, α4-1BB VS α4-1BB/AlloDC P=0.0235) (**Figure 3K**).

Our previous study indicated that AlloDCs exert immune-priming effects through the activation of host DCs, and thus we further characterized the tumor-infiltrated DCs as well. When analyzing the recruited tumor-infiltrated DC population, there was no difference in the number of infiltrating host DCs between the combination treatment group and their corresponding monotherapies (**Figure 3L**). However, in the combination treatment group, DCs were featured with high antigen-presenting capacity with high CD103 (PBS VS α4-1BB/AlloDC: P=0.0127), high CD8a expression (PBS VS α4-1BB/AlloDC: P=0.0131) (**Figures 3M, N**), and high CCL5 (PBS VS α4-1BB/AlloDC: P=0.0420) and IL-12 secretion (PBS VS α4-1BB/AlloDC: P=0.0415) (**Figures 3O, P**).

Taken together, adding AlloDC to α4-1BB therapy enhances the antigen-presenting capacity and activation state of host DCs, which might contribute to unleashing the anti-tumor CD8 T-cell response. This is also in line with our previous finding that AlloDC injected as an adjuvant created a proinflammatory environment, which further recruits and activates host antigen-presenting DCs (7).

### 3.4 AlloDCs combined with α4-1BB treatment enhance infiltration of myeloid lineage cells with less suppressive phenotypes

**Figure 4:**
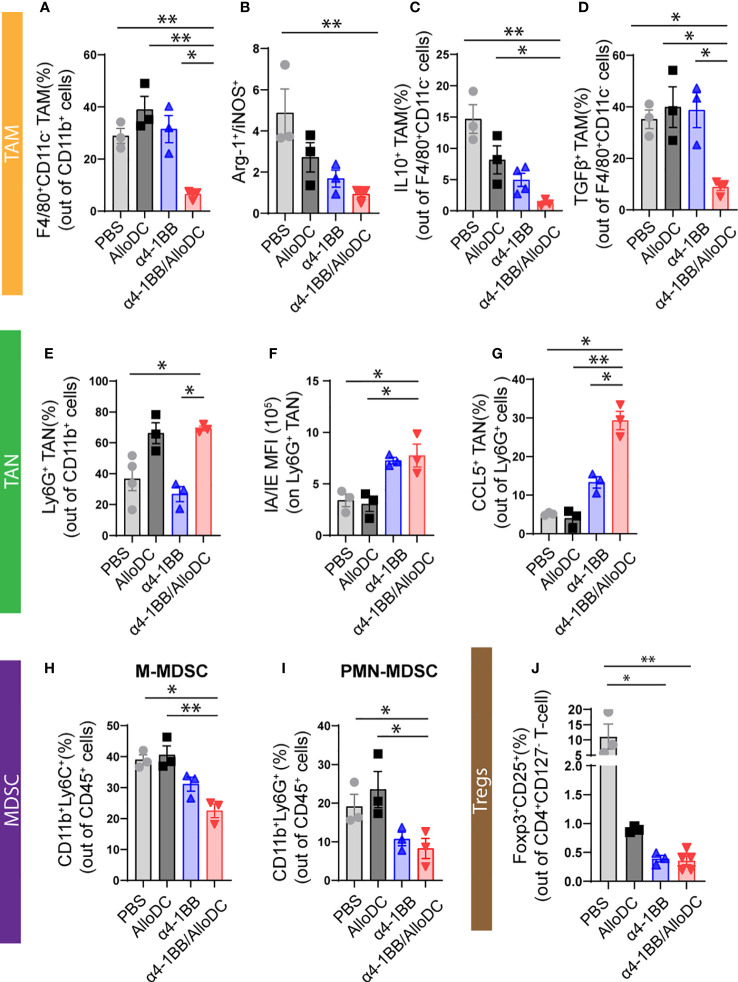
AlloDCs combined with α4-1BB treatment enhance infiltration of immune cells with less suppressive phenotypes. **(A)**Percentage of the tumor-associated macrophages (TAM, F4/80^+^ CD11C^-^) in each treatment group. **(B)** The ratio between either Arginase-I^+^ or iNOS^+^ TAM. **(C, D)** Percentage of the IL10^+^ **(C)** and TGFβ^+^ **(D)** TAM. **(E)** Percentage of the tumor-associated neutrophils (TAN, Ly6G^+^ cells) in each treatment group. **(F)** MFI of MHC-II IA/IE on TAN. **(G)** Percentage of the CCL5^+^ TAN in each treatment group. **(H–J)** Percentage of M-MDSC **(H)**, PMN-MDSC **(I)**, and CD4^+^ Treg **(J)** in the tumor. Error bars represent SEM and the mean values were compared using one-way ANOVA nonparametric test. (*P<0.05, **P<0.01).

Our previous study indicated that AlloDCs shape the TME to a less suppressive anti-tumoral environment (1, 2), thus the tumor-infiltrating myeloid cells were characterized. Firstly, although α4-1BB/AlloDC combination treatment group presented significantly fewer tumor-infiltrating macrophages (PBS VS α4-1BB/AlloDC: P=0.0063) (**Figure 4A**), they expressed lower Arginase-I and higher iNOS (PBS VS α4-1BB/AlloDC: P=0.0066) (**Figure 4B**), indicating an M1-like phenotype. Moreover, IL-10 and TGF-β secreting macrophages were notably reduced in the combination treatment group (IL-10: PBS VS α4-1BB/AlloDC: P=0.0024; TGF-β; PBS VS α4-1BB/AlloDC: P=0.0499) (**Figures 4C, D**). There was also enhanced infiltration of neutrophils with higher IA/IE expression TGF-β (PBS VS α4-1BB/AlloDC: P=0.0356) (**Figures 4E, F**) and higher CCL5 secretion (PBS VS α4-1BB/AlloDC: P=0.0174) (**Figure 4G**) in the α4-1BB/AlloDC combination treatment group. On the other hand, the groups receiving α4-1BB alone or in combination exhibited reduced infiltration of monocytic myeloid-derived suppressor cells (M-MDSC) (PBS VS α4-1BB/AlloDC: P=0.0174), polymorphonuclear myeloid-derived suppressor cells (PMN-MDSC) (PBS VS α4-1BB/AlloDC: P=0.0416), and CD4^+^ Tregs (PBS VS α4-1BB/AlloDC: P=0.0048) (**Figures 4H–J**).

Taken together, these data suggest that adding intratumoral administration of AlloDCs to systemic α4-1BB therapy enhances intratumoral infiltration of immune cells to generate an immune-inflamed and less-suppressive TME.

## 4 Discussion

Ligation of 4-1BB and its ligand has been found to be important for T cell functions by increasing its proliferation (19), enhancing cytokines production (19), reducing cell apoptosis (20), polarizing memory differentiation (21), and reverting anergy/exhaustion (22). Agonistic antibodies targeting 4-1BB showed promising antitumor effects in various pre-clinical models and were reported to be CD8 T-cell dependent (11, 23). While the clinical development of therapeutic antibodies targeting 4-1BB, the human IgG4 antibody Urelumab exhibited liver toxicity (24). The side effects can be mitigated with dose reduction; however, this also restricted its clinical activity (25). On the other hand, the human IgG2 antibody Utomilumab, despite showing excellent tolerability and safety, clinical activity was as modest as a monotherapy (26). These results clearly suggested that one object of developing 4-1BB agonistic antibody-based approaches is to finetune the treatment regimen that can retain clinically meaningful agonistic activity with tolerable toxicity, either as a monotherapy or in combination with other treatments.

Given the fact that 4-1BB is largely expressed on antigen-experienced T cells and agonistic 4-1BB antibody exert its function largely rely on T cells, we thus in this study investigated whether the therapeutic efficacy of agonistic 4-1BB antibody could be enhanced by adding a new mode-of-action, the T cell priming using AlloDC. We hypothesize that with an enhanced T cell priming, the anti-tumor efficacy of α4-1BB might be provoked at its tolerable dosage.

AlloDCs have been proven to trigger a proinflammatory milieu potentially reverting the immune-suppressive TME due to an allogeneic reaction (2). Subsequently, AlloDCs will prime a T cell response by activating recruited endogenous DCs, which induces an anti-cancer response (2). Our previous finding indicated that AlloDCs could efficiently modulate the TME and increase T-cell infiltration, even though they failed to control tumor growth as a monotherapy. Notably, when AlloDCs was combined with immune checkpoint inhibitor αCTLA-4, a strong anti-tumor synergy mediated by enhanced antigen-specific and tissue-resident memory T cells response was observed (7). Instead of αCTLA-4 which blocks the inhibitory checkpoints expressed on regulatory T-cells, in this study, α4-1BB was combined with AlloDCs to directly potentiate the effector cells response. And AlloDCs, in the combined treatment, serve as a stimulus to augment the T cells response, in order to enhance the therapeutic efficiency.

Our data show that α4-1BB monotherapy exhibited a therapeutic effect against CT-26 tumors which has also been observed in several other tumor models and can be attributed to the direct stimulation of 4-1BB on effector cells (11) and blocking of the reverse signaling via 4-1BBL (27). Notably, the addition of AlloDC to α4-1BB therapy significantly increased the curative rate in tumor-bearing mice from 35% to 70%. Our concept of combination therapy also showed therapeutic benefit against B16 tumors which indicates AlloDCs as a general intratumoral immune adjuvant since their effect were not restricted to a certain mouse strain.

The synergistic benefit can be explained by AlloDC providing an overall proinflammatory environment and eliciting an effective T cell response (**Figures 2C–E**). In the α4-1BB/AlloDC combination group, we observed an overall higher infiltration of CD8 T-cells (**Figures 2F**, **3A, B**), especially T_RM_ (**Figures 3H, I**), which was shown to control tumor growth by producing granzyme B and IFN-γ for the direct killing of tumors, eliciting the production of chemokines and expression of adhesion molecules (28). The level of CD107a^+^ on CD8^+^ T-cell were similar across different groups, probably due to the time of assessment, as IFN-γ^+^CD8^+^ T cells were clearly increased in the combination therapy group indicating T cell toxicity. The increase of CD69^+^ CD8 T cells in tumor-draining lymph node in the combination group also confirmed that AlloDCs can initiate the T cells response. CD4 depletion didn’t abolish the efficacy of α4-1BB/AlloDC treatment, indicating that the agonistic α4-1BB antibody can directly potentiate the response of primed CD8 T cells without help from CD4 T cells. Moreover, more activated intratumoral DCs with high antigen-presenting ability were found in the α4-1BB/AlloDCs group, in line with a report that agonistic 4-1BB signaling induces DC maturation, IL-12 secretion, and enhances antigen presentation capacity (29). Skewing of tumor-infiltrating macrophages towards a M1 profile was also observed which is in accordance with a study showing that stimulation of macrophages with 4-1BB can enhance their capacity for antigen presentation (30). Intratumoral neutrophils were also polarized towards N1 phenotype with high IA/IE expression and CCL5 secretion (**Figures 4E–G**), similar to what was describe in the study of agonistic 4-1BB antibodies (31). CCL5, a potent chemoattractant, plays an important role in attracting T cells, NK cells, macrophages, and immature dendritic cells (32). CCL5 was strongly expressed in tumor-infiltrating DCs and neutrophils in the α4-1BB/AlloDCs group, which may explain the high infiltration of certain immune cells in the TME. Lastly, the combination treatment resulted in reduced infiltration of M-MDSC, PMN-MDSC and Tregs. Although the α4-1BB stimulation of regulatory cells is controversial (9), less suppressive immune cell infiltration was observed in our combination groups. Further confirmation is needed to determine whether the α4-1BB agonist inhibits the differentiation of conventional effector cells into Tregs and also suppresses Treg activity.

Combination therapy of agonistic 4-1BB antibodies has been tested clinically with PD-(L)1 checkpoint inhibitors. The contribution of 4-1BB antibodies is hard to conclude in both the case of urelumab + nivolumab (24) and utomilumab + pembrolizumab (33) combinations due to lack of comparison arm. However, historical data suggest PD-(L)1 checkpoint inhibitors as monotherapy is active across multiple malignancies with response ranging from 10-30%, the combination therapy did not show any major improvements (34). One speculation could be that PD-(L)1 checkpoint inhibitors and 4-1BB antibodies both rely on the function of pre-existing T cells response. In contrast, AlloDCs function as an immune primer which can initiate the T cell response, and the α4-1BB antibody can further boost the efficacy of these primed cells.

In conclusion, we present data showing that AlloDCs can synergistically enhance therapeutic efficacy of systemic treatment with agonistic α4-1BB antibodies. When α4-1BB is combined with AlloDCs, the treatment altered the immunosuppressive microenvironment with enhanced infiltration of matured and antigen-presenting tumor-infiltrating DCs and reduced number of MDSC and Tregs, which supports the effectiveness of cytotoxic CD8 T-cells. This inhibition of local immunosuppression may explain the decreased signs of exhaustion in CD8 T-cells and an increase in the T_RM_ cells. Such scenario would further increase the number of tumor-specific T-cells and thus maintains the cancer immunity cycle to generate long-term protection. Therefore, AlloDCs serves as a promising immune-priming candidate for therapeutic use that can amplify the anti-tumor immunity of agonistic antibody therapy leading to increased tumor response rates and potential cure.

## Data availability statement

The original contributions presented in the study are included in the article/**Supplementary Materials**, further inquiries can be directed to the corresponding author/s.

## Ethics statement

The animal study was reviewed and approved by The Northern Stockholm Research Animal Ethics Committee has approved the animal studies (5.8.18-19434/2019).

## Author contributions

CJ, DY and AK designed the experiments. CJ, AA, MH and AI performed the experiments and analyzed the data. CJ, MH and DY, wrote the paper, and HW and AK revised the paper. All authors contributed to the article and approved the submitted version.

## Supplementary material

Supplementary Figure 1 Percentage of CD8^+^ T cells **(A)** and CD69^+^ CD8 T cells **(B)** in draining lymph nodes from different treatment groups. (*: P<0.05, **: P<0.01, ****: P<0.0001).

## References

[1] Fotaki G Jin C Kerzeli IK Ramachandran M Martikainen MM Karlsson-Parra A. Cancer vaccine based on a combination of an infection-enhanced adenoviral vector and pro-inflammatory allogeneic DCs leads to sustained antigen-specific immune responses in three melanoma models. Oncoimmunology (2018) 7(3):e1397250. doi: 10.1080/2162402X.2017.1397250 29399398 PMC5790347

[2] Fotaki G Jin C Ramachandran M Kerzeli IK Karlsson-Parra A Yu D. Pro-inflammatory allogeneic DCs promote activation of bystander immune cells and thereby license antigen-specific T-cell responses. Oncoimmunology (2018) 7(3):e1395126. doi: 10.1080/2162402X.2017.1395126 29399392 PMC5790348

[3] Rizell M Sternby Eilard M Andersson M Andersson B Karlsson-Parra A Suenaert P. Phase 1 trial with the cell-based immune primer ilixadencel, alone, and combined with sorafenib, in advanced hepatocellular carcinoma. Front Oncol (2019) 9:19. doi: 10.3389/fonc.2019.00019 30719425 PMC6348253

[4] Frobom R Berglund E Berglund D Nilsson IL Ahlen J von Sivers K. Phase I trial evaluating safety and efficacy of intratumorally administered inflammatory allogeneic dendritic cells (ilixadencel) in advanced gastrointestinal stromal tumors. Cancer Immunol Immunother (2020) 69(11):2393–401. doi: 10.1007/s00262-020-02625-5 PMC7568699 32535637

[5] Karlsson-Parra A Kovacka J Heimann E Jorvid M Zeilemaker S Longhurst S. Ilixadencel - an allogeneic cell-based anticancer immune primer for intratumoral administration. Pharm Res (2018) 35(8):156. doi: 10.1007/s11095-018-2438-x 29904904 PMC6002422

[6] Lindskog M Laurell A Kjellman A Melichar B Rey PM Zielinski H. Ilixadencel, a cell-based immune primer, plus sunitinib versus sunitinib alone in metastatic renal cell carcinoma: A randomized phase 2 study. Eur Urol Open Sci (2022) 40:38–45. doi: 10.1016/j.euros.2022.03.012 35638086 PMC9142735

[7] Jin C Ali A Iskantar A Fotaki G Wang H Essand M. Intratumoral administration of pro-inflammatory allogeneic dendritic cells improved the anti-tumor response of systemic anti-CTLA-4 treatment via unleashing a T cell-dependent response. Oncoimmunology (2022) 11(1):2099642. doi: 10.1080/2162402X.2022.2099642 35859733 PMC9291714

[8] Pardee AD Wesa AK Storkus WJ. Integrating costimulatory agonists to optimize immune-based cancer therapies. Immunotherapy (2009) 1(2):249–64. doi: 10.2217/1750743X.1.2.249 PMC2746690 20046961

[9] Makkouk A Chester C Kohrt HE. Rationale for anti-CD137 cancer immunotherapy. Eur J Cancer (2016) 54:112–9. doi: 10.1016/j.ejca.2015.09.026 26751393

[10] Chester C Sanmamed MF Wang J Melero I. Immunotherapy targeting 4-1BB: mechanistic rationale, clinical results, and future strategies. Blood (2018) 131(1):49–57. doi: 10.1182/blood-2017-06-741041 29118009

[11] Miller RE Jones J Le T Whitmore J Boiani N Gliniak B. 4-1BB-specific monoclonal antibody promotes the generation of tumor-specific immune responses by direct activation of CD8 T cells in a CD40-dependent manner. J Immunol (2002) 169(4):1792–800. doi: 10.4049/jimmunol.169.4.1792 12165501

[12] Tirapu I Arina A Mazzolini G Duarte M Alfaro C Feijoo E. Improving efficacy of interleukin-12-transfected dendritic cells injected into murine colon cancer with anti-CD137 monoclonal antibodies and alloantigens. Int J Cancer (2004) 110(1):51–60. doi: 10.1002/ijc.20093 15054868

[13] Westwood JA Haynes NM Sharkey J McLaughlin N Pegram HJ Schwendener RA. Toll-like receptor triggering and T-cell costimulation induce potent antitumor immunity in mice. Clin Cancer Res (2009) 15(24):7624–33. doi: 10.1158/1078-0432.CCR-09-2201 19996209

[14] Molckovsky A Siu LL. First-in-class, first-in-human phase I results of targeted agents: highlights of the 2008 American society of clinical oncology meeting. J Hematol Oncol (2008) 1:20. doi: 10.1186/1756-8722-1-20 18959794 PMC2647552

[15] Bartkowiak T Singh S Yang G Galvan G Haria D Ai M. Unique potential of 4-1BB agonist antibody to promote durable regression of HPV+ tumors when combined with an E6/E7 peptide vaccine. Proc Natl Acad Sci U S A (2015) 112(38):E5290–9. doi: 10.1073/pnas.1514418112 PMC4586868 26351680

[16] Enamorado M Iborra S Priego E Cueto FJ Quintana JA Martinez-Cano S. Enhanced anti-tumour immunity requires the interplay between resident and circulating memory CD8(+) T cells. Nat Commun (2017) 8:16073. doi: 10.1038/ncomms16073 28714465 PMC5520051

[17] Duhen T Duhen R Montler R Moses J Moudgil T de MIranda NF. Co-expression of CD39 and CD103 identifies tumor-reactive CD8 T cells in human solid tumors. Nat Commun (2018) 9(1):2724. doi: 10.1038/s41467-018-05072-0 30006565 PMC6045647

[18] Kalos M. Tumor antigen-specific T cells and cancer immunotherapy: current issues and future prospects. Vaccine (2003) 21(7-8):781–6. doi: 10.1016/S0264-410X(02)00598-4 12531359

[19] Pollok KE Kim YJ Zhou Z Hurtado J Kim KK Pickard RT. Inducible T cell antigen 4-1BB. Analysis of expression and function. J Immunol (1993) 150(3):771–81. 7678621

[20] Hurtado JC Kim YJ Kwon BS. Signals through 4-1BB are costimulatory to previously activated splenic T cells and inhibit activation-induced cell death. J Immunol (1997) 158(6):2600–9. doi: 10.4049/jimmunol.158.6.2600 9058792

[21] Zhou AC Wagar LE Wortzman ME Watts TH. Intrinsic 4-1BB signals are indispensable for the establishment of an influenza-specific tissue-resident memory CD8 T-cell population in the lung. Mucosal Immunol (2017) 10(5):1294–309. doi: 10.1038/mi.2016.124 28051085

[22] Williams JB Horton BL Zheng Y Duan Y Powell JD Gajewski TF. The EGR2 targets LAG-3 and 4-1BB describe and regulate dysfunctional antigen-specific CD8+ T cells in the tumor microenvironment. J Exp Med (2017) 214(2):381–400. doi: 10.1084/jem.20160485 28115575 PMC5294847

[23] Melero I Shuford WW Newby SA Aruffo A Ledbetter JA Hellstrom KE. Monoclonal antibodies against the 4-1BB T-cell activation molecule eradicate established tumors. Nat Med (1997) 3(6):682–5. doi: 10.1038/nm0697-682 9176498

[24] Segal NH Logan TF Hodi FS McDermott D Melero I Hamid O. Results from an integrated safety analysis of urelumab, an agonist anti-CD137 monoclonal antibody. Clin Cancer Res (2017) 23(8):1929–36. doi: 10.1158/1078-0432.CCR-16-1272 27756788

[25] Timmerman J Herbaux C Ribrag V Zelenetz AD Houot R Neelapu SS. Urelumab alone or in combination with rituximab in patients with relapsed or refractory B-cell lymphoma. Am J Hematol (2020) 95(5):510–20. doi: 10.1002/ajh.25757 PMC7383599 32052473

[26] Segal NH He AR Doi T Levy R Bhatia S Pishvaian MJ. Phase I study of single-agent utomilumab (PF-05082566), a 4-1BB/CD137 agonist, in patients with advanced cancer. Clin Cancer Res (2018) 24(8):1816–23. doi: 10.1158/1078-0432.CCR-17-1922 29549159

[27] Kang SW Lee SC Park SH Kim J Kim HH Lee HW. Anti-CD137 suppresses tumor growth by blocking reverse signaling by CD137 ligand. Cancer Res (2017) 77(21):5989–6000. doi: 10.1158/0008-5472.CAN-17-0610 28923858

[28] Amsen D van Gisbergen K Hombrink P van Lier RAW. Tissue-resident memory T cells at the center of immunity to solid tumors. Nat Immunol (2018) 19(6):538–46. doi: 10.1038/s41590-018-0114-2 29777219

[29] Choi BK Kim YH Kwon PM Lee SC Kang SW Kim MS. 4-1BB functions as a survival factor in dendritic cells. J Immunol (2009) 182(7):4107–15. doi: 10.4049/jimmunol.0800459 PMC2681223 19299708

[30] Stoll A Bruns H Fuchs M Völkl S Nimmerjahn F Kunz M. CD137 (4-1BB) stimulation leads to metabolic and functional reprogramming of human monocytes/macrophages enhancing their tumoricidal activity. Leukemia (2021) 35(12):3482–96. doi: 10.1038/s41375-021-01287-1 PMC8632678 34021248

[31] Lee SC Ju SA Sung BH Heo SK Cho HR Lee EA. Stimulation of the molecule 4-1BB enhances host defense against Listeria monocytogenes infection in mice by inducing rapid infiltration and activation of neutrophils and monocytes. Infect Immun (2009) 77(5):2168–76. doi: 10.1128/IAI.01350-08 PMC2681750 19237524

[32] Appay V Rowland-Jones SL. RANTES: a versatile and controversial chemokine. Trends Immunol (2001) 22(2):83–7. doi: 10.1016/S1471-4906(00)01812-3 11286708

[33] Tolcher AW Sznol M Hu-Lieskovan S Papadopoulos KP Patnaik A Rasco DW. Phase ib study of utomilumab (PF-05082566), a 4-1BB/CD137 agonist, in combination with pembrolizumab (MK-3475) in patients with advanced solid tumors. Clin Cancer Res (2017) 23(18):5349–57. doi: 10.1158/1078-0432.CCR-17-1243 28634283

[34] Pennock GK Chow LQ. The evolving role of immune checkpoint inhibitors in cancer treatment. Oncologist (2015) 20(7):812–22. doi: 10.1634/theoncologist.2014-0422 PMC4492230 26069281

