# Supplementary material for: Proinflammatory allogeneic dendritic cells enhance the therapeutic efficacy of systemic anti-4-1BB treatment
Source: Front Immunol. 2023 Aug 15;14:1146413. doi: 10.3389/fimmu.2023.1146413 (PMC10466132; doi:10.3389/fimmu.2023.1146413)
Supplement: Supplementary file 1 [file Table_1.docx]

| **Table S1. Antibodies used in the current study** |  |  |  |  |  |
| --- | --- | --- | --- | --- | --- |
| **Experiment** | **Antibody** | **Color** | **Cat number** | **Clone** | **Company** |
| **Mouse tumor myeloid panel** | CD11b | BUV395 | 563553 | M1/70 | BD bioscience |
|  | CD86 | BUV737 | 741737 | GL1 | BD bioscience |
|  | TGF-β | BV421 | 565638 | TW7-16B4 | BD bioscience |
|  | CD45 | BV510 | 563891 | 30-F11 | BD bioscience |
|  | Ly6G | BV605 | 563005 | 1AB | BD bioscience |
|  | PDL1 | BV650 | 124336 | 10G.9G2 | Biolegend |
|  | CD103 | BV711 | 564320 | M290 | BD bioscience |
|  | Ly6C | BV786 | 128041 | HK1.4 | Biolegend |
|  | iNOS | BB515 | 53-5920-82 | CXNFT | ebioscience |
|  | IA-IE | BB700 | 746197 | M5/114.15.2 | BD bioscience |
|  | CCL5 | PE | 149103 | 2E9/CCL5 | Biolegend |
|  | IL10 | PE-CF594 | 505033 | JES5-16E3 | Biolegend |
|  | F4/80 | PE-Cy5 | 123111 | BM8 | Biolegend |
|  | CD8a | PE-Cy5.5 | 100791 | 53-6.7 | Biolegend |
|  | Arginase-I | PE-Cy7 | 25-3697-82 | A1exF5 | ebioscience |
|  | IL-12(p40/p70) | APC | 554480 | C15.6 | BD bioscience |
|  | CD11c | APC-Cy7 | 561039 | N418 | BD bioscience |
|  | Fixable Viability Stain | FVS700 | 565388 |  | BD bioscience |
| **Mouse tumor T cell panel** | TCRb | BUV395 | 742485 | H57-97 | BD bioscience |
|  | CD4 | BUV496 | 612952 | GK1.5 | BD bioscience |
|  | CD8 | BUV737 | 612759 | 53-6.7 | BD bioscience |
|  | CD107 | BV421 | 564347 | 1D4B | BD bioscience |
|  | CD45 | BV510 | 563891 | 30-F11 | BD bioscience |
|  | Tim-3 | BV605 | 119721 | RMT3-23 | Biolegend |
|  | LAG-3 | BV711 | 563179 | C9B7W | BD bioscience |
|  | PD-1 | BV786 | 123225 | 29F.1A12 | Biolegend |
|  | CD44 | BB515 | 564587 | IM7 | BD bioscience |
|  | CD69 | BB700 | 566500 | H1.2F3 | BD bioscience |
|  | IFN-γ | PE | 562020 | XMG1.2 | BD bioscience |
|  | CXCR6 | PE-CF594 | 151116 | SA051D1 | Biolegend |
|  | CD127 | PE-Cy5 | 125016 | A7R34 | Biolegend |
|  | CD62L | PE-Cy7 | 565535 | MEL-14 | BD bioscience |
|  | ICOS | APC | 107711 | 15F9 | Biolegend |
|  | Fixable Viability Stain | FVS700 | 565388 |  | BD bioscience |
| **Mouse tumor tissue-residence T cell panel** | TCRb | BUV395 | 742485 | H57-97 | BD bioscience |
|  | CD4 | BUV496 | 612952 | GK1.5 | BD bioscience |
|  | CD8 | BUV737 | 612759 | 53-6.7 | BD bioscience |
|  | CD45 | BV510 | 563891 | 30-F11 | BD bioscience |
|  | CD103 | BV711 | 564320 | M290 | BD bioscience |
|  | CCR7 | BB700 | 566464 | 4B12 | BD bioscience |
|  | CD49a | PE | 142603 | HMa1 | Biolegend |
|  | CD127 | PE-Cy5 | 125016 | A7R34 | Biolegend |
|  | CD39 | PE-Cy7 | 565535 | 15F9 | BD bioscience |
|  | CD69 | APC | 560689 | H1.2F3 | BD bioscience |
|  | Fixable Viability Stain | FVS700 | 565388 |  | BD bioscience |
| **Mouse exhuasted T cell panel** | TCRb | BUV395 | 742485 | H57-97 | BD bioscience |
|  | CD4 | BUV496 | 612952 | GK1.5 | BD bioscience |
|  | CD8 | BUV737 | 612759 | 53-6.7 | BD bioscience |
|  | Tim-3 | BV605 | 119721 | RMT3-23 | Biolegend |
|  | LAG-3 | BV711 | 563179 | C9B7W | BD bioscience |
|  | PD-1 | BV786 | 123225 | 29F.1A12 | Biolegend |
|  | Fixable Viability Stain | FVS700 | 565388 |  | BD bioscience |
| **Mouse Treg panel** | TCRb | BUV395 | 742485 | H57-97 | BD bioscience |
|  | CD4 | BUV496 | 612952 | GK1.5 | BD bioscience |
|  | IL10 | PE-CF594 | 505033 | JES5-16E3 | Biolegend |
|  | CD25 | PE | 553075 | 3C7 | BD bioscience |
|  | Foxp3 | APC | 320013 | 150D | Biolegend |
|  | CD127 | PE-Cy5 | 125016 | A7R34 | Biolegend |
|  | CD45 | BV510 | 563891 | 30-F11 | BD bioscience |
|  | Fixable Viability Stain | FVS700 | 565388 |  | BD bioscience |
| **Mouse MDSC panel** | CD45 | BV510 | 563891 | 30-F11 | BD bioscience |
|  | CD11b | BUV395 | 563553 | M1/70 | BD bioscience |
|  | Ly6G | BV605 | 563005 | 1AB | BD bioscience |
|  | Ly6C | BV786 | 128041 | HK1.4 | Biolegend |
|  | CD11c | APC-Cy7 | 561039 | N418 | BD bioscience |
|  | Fixable Viability Stain | FVS700 | 565388 |  | BD bioscience |
