# Supplementary figures and images for: Proinflammatory allogeneic dendritic cells enhance the therapeutic efficacy of systemic anti-4-1BB treatment
Source: Front Immunol. 2023 Aug 15;14:1146413. doi: 10.3389/fimmu.2023.1146413 (PMC10466132; doi:10.3389/fimmu.2023.1146413)

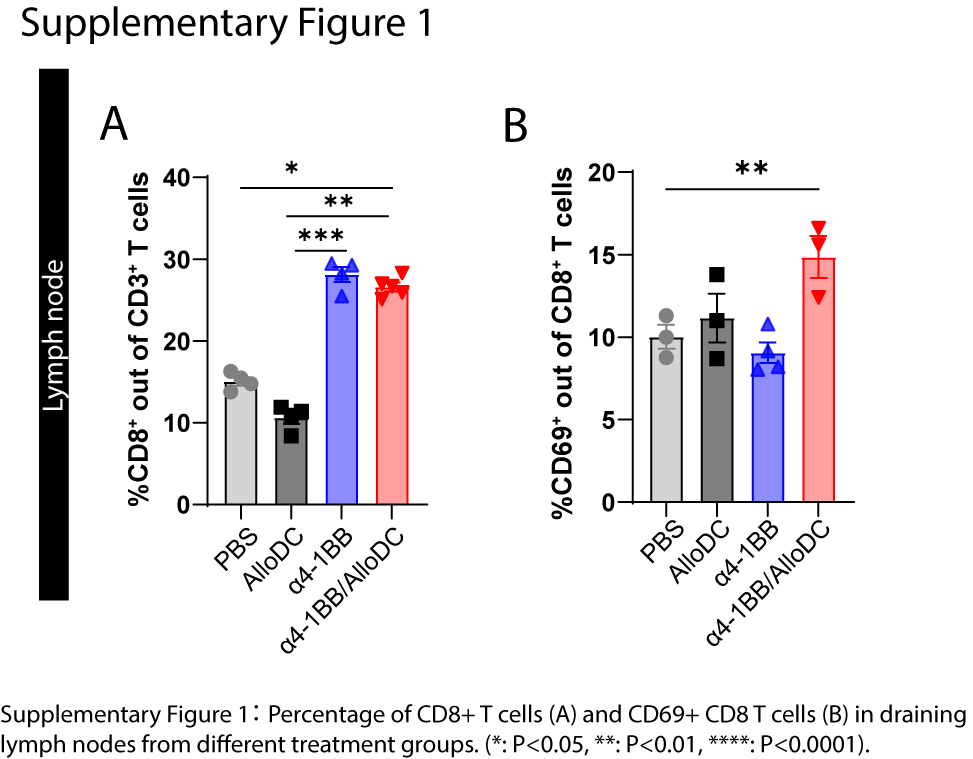

Supplement: Supplementary Figure 1 — Percentage of CD8+ T cells (A) and CD69+ CD8 T cells (B) in draining lymph nodes from different treatment groups. (*: P<0.05, **: P<0.01, ****: P<0.0001). [file Image_1.tif]
